# Cytoreductive surgery and hyperthermic intraperitoneal chemotherapy (HIPEC) for colorectal peritoneal metastases: analysis of short- and long-term outcomes

**DOI:** 10.1007/s00423-021-02353-z

**Published:** 2021-10-18

**Authors:** Fausto Rosa, Federica Galiandro, Riccardo Ricci, Dario Di Miceli, Giuseppe Quero, Claudio Fiorillo, Caterina Cina, Sergio Alfieri

**Affiliations:** 1 Department of Digestive Surgery, Fondazione Policlinico Universitario Agostino Gemelli IRCCS, Largo A. Gemelli, 8, 00168 Rome, Italy; 2 Università Cattolica del Sacro Cuore, Rome, Italy; 3 Department of Pathology, Fondazione Policlinico Universitario Agostino Gemelli IRCCS, Rome, Italy; 4 General Surgery, Ospedale Buccheri-La Ferla, Palermo, Italy

**Keywords:** Colorectal cancer, Cytoreductive surgery, HIPEC, Morbidity, Mortality

## Abstract

**Background:**

Peritoneal metastases carry the worst prognosis among all sites of colorectal cancer (CRC) metastases. In recent years, the advent of cytoreductive surgery (CRS) and hyperthermic intraperitoneal chemotherapy (HIPEC) has improved survival for selected patients with limited peritoneal involvement. We report the evolution of CRS and HIPEC for colorectal peritoneal metastases at a tertiary referral center over a 10-year period.

**Methods:**

Patients with colorectal peritoneal metastases undergoing CRS and HIPEC were included and retrospectively analyzed at a tertiary referral center from January 2006 to December 2015. Main outcomes included evaluation of grade III/IV complications, mortality rate, overall and disease-free survival, and prognostic factors influencing survival on a Cox multivariate analysis.

**Results:**

Sixty-seven CRSs were performed on 67 patients during this time for colorectal peritoneal metastases. The median patient age was 57 years with 55.2% being female. The median peritoneal carcinomatosis index (PCI) was 7, with complete cytoreduction achieved in 65 (97%) cases. Grade > 2 complications occurred in 6 cases (8.9%) with no mortality. The median overall survival for the entire cohort was 41 months, with a 3-year overall survival of 43%. In case of complete cytoreduction, median overall and disease-free survival were 57 months and 36 months respectively, with a 3-year disease-free survival of 62%. Complete cytoreduction and nonmucinous histology were key factors independently associated with improved overall survival.

**Conclusions:**

CRS and HIPEC for limited peritoneal metastases from CRC are safe and effective, with acceptable morbidity. In selected patients, it offers a highly favorable long-term outcomes.

## Introduction

Colorectal cancer (CRC) represents the third most common cancer, and the second cause of cancer-related mortality all around the world [1]. About half of CRC patients will develop metastases, and the peritoneum represents the third most common site and is associated with the worst prognosis among all metastatic sites [2]. Synchronous peritoneal metastases are seen in 5 to 7% of cases of CRC [3], whereas metachronous disease can involve up to 19% of cases [3, 4].

Patients suffering from peritoneal metastases from CRC present a median survival of less than 6 months [5].

In highly selected patients, the association of cytoreductive surgery (CRS) and hyperthermic intraperitoneal chemotherapy (HIPEC) can offer the possibility of long-term survival.

Initially proposed and diffused by Sugarbaker [6–8] for the treatment of disseminated appendiceal neoplasms, CRS and HIPEC have since been expanded to cases of peritoneal metastasis from CRC and other abdominal neoplasms. In recent years, specialized centers in peritoneal surface malignancies have reported highly favorable outcomes with a median overall survival ranging from 22 to 63 months and a 5-year survival from 19 to 51% in selected patients with isolated peritoneal involvement [9–11].

CRS and HIPEC presented a wide diffusion all around the world after a consensus statement that strongly supported their use in 2007 [12]. However, there are some concerns regarding the utility of CRS and HIPEC and the associated morbidity.

The aim of our study was to evaluate the safety and efficacy of CRS and HIPEC for peritoneal metastases from CRC at a tertiary care center, and to assess the related short- and long-term outcomes.

## Methods

Data were retrospectively analyzed from the prospectively collected database on peritoneal surface malignancies. All patients undergoing CRS and HIPEC for peritoneal metastases from CRC from January 1, 2006, to December 31, 2015, were included (20 patients from January 1, 2006, to December 31, 2009, and 47 patients from January 1, 2010, to December 31, 2015).

Institutional review board approval had been preliminarily obtained for the research purpose use of the data, stemming out from standard clinical practice, since no additional interventions were planned (observational study).

Patient age, sex, ASA score, primary tumor location, histopathology details, and perioperative chemotherapy use were recorded.

### Inclusion/exclusion criteria

All patients were preoperatively assessed with a full history and examination. Routine full blood count, CEA, liver function tests, and coagulation studies, along with an FDG-PET/CT scan of the chest, abdomen, and pelvis, were performed.

All patient cases were reviewed at the colorectal cancer multidisciplinary meeting.

Inclusion criteria were as follows: age 18–80 years; normal cardiac, respiratory, liver, and renal functions; and no hematological alterations.

Exclusion criteria for HIPEC were uncontrolled severe infection and/or medical problems unrelated to malignancy which would limit full compliance with the protocol or expose the patient to extreme risk of life.

Patients with concurrent extra-abdominal disease or very high–volume disease on imaging were not offered CRS and HIPEC.

Those with clearly very low–volume disease on imaging were offered CRS and HIPEC.

Preoperative peritoneal carcinomatosis index (PCI) was evaluated in all patients through a contrast-enhanced CT scan. Diagnostic laparoscopy was selectively performed if there was moderate-volume disease on imaging.

The policy of our local multidisciplinary team was to offer CRS and HIPEC to patients with a PCI of 25 or below.

The same inclusion/exclusion criteria were followed for patients undergoing neoadjuvant chemotherapy.

### Operative details and HIPEC

Following laparotomy and adhesiolysis, an assessment of the PCI was conducted according to the Sugarbaker evaluation [6]. This was scored from 0 to 39.

CRS was performed in keeping with the Sugarbaker techniques [7, 8], as directly learned by the senior surgeon (S.A.) during his fellowship at Washington Cancer Center. Organ resections involved resection of the involved regions; resection of the so-called target regions even in the absence of visible disease (lesser omentum, gallbladder, falciform, and umbilical round ligaments); and omental resection, including gastroepiploic arch in the presence of visible disease. Peritonectomy was performed removing all peritoneum involved. The completeness of cytoreduction score (CC score) was recorded at the end of each operation. CC-0 reflected no remaining visible disease. CC-1, CC-2, and CC-3 implied remaining disease less than 2.5 mm, 2.5 to 2.5 cm, and greater than 2.5 cm.

HIPEC was administered only when a complete cytoreduction was achieved. The HIPEC procedure was administered for 90 min with an inflow temperature of 41–42 °C and an outflow temperature of 39–40 °C, using mitomycin C (MMC) at a dose of 15 mg/m^2^.

### Complications

Major complications (grade III and IV) were recorded according to the Clavien-Dindo classification system [13].

### Statistical analysis

All results were expressed as median with an interquartile range for continue variables, and absolute and percentage frequencies for categorical variables.

Follow-up was analyzed with the reverse Kaplan–Meier method. Survival curves were estimated by the Kaplan–Meier product limit method.

To identify factors independently associated with overall survival, a Cox univariate and multivariate hazards ratio model was used. A *p* value of < 0.05 was considered statistically significant.

Statistical analyses were performed using SPSS 20.0 (IBM Corp. SPSS Statistics).

## Results

### Demographic and operative data

Sixty-seven patients underwent 67 cytoreductive surgeries during the time period of the study.

Demographic characteristics of patients and histopathological and molecular characteristics of the tumors are shown in Table 1.

**Table 1:** Demographic characteristics of patients and histopathological characteristics of the tumors in 67 patients

HIPEC procedures, *n*	67
Age, *years, median (range)*	57 (27–82)
Sex	
Female, *n (%)*	37 (55.2)
Male, *n (%)*	30 (44.8)
Primary tumor location	
Right colon*, n* (%)	36 (53.7)
Transverse colon, *n* (%)	4 (5.9)
Left colon, *n* (%)	27 (34.5)
Neoadjuvant chemotherapy, *n (%)*	6 (8.9)
Histological findings	
Adenocarcinoma, *n (%)*	42 (62.7)
Mucinous adenocarcinoma, *n (%)*	25 (37.3)
Degree of tumor differentiation	
G1	11
G2	33
G3	23
pT stage	
T2	4 (6)
T3	18 (26.9)
T4a	29 (43.2)
T4b	16 (23.9)
pN stage	
N0	24 (35.8)
N1	33 (49.2)
N1a	11
N1b	13
N1c	9
N2	10 (15)
N2a	7
N2b	3
RAS*	
Mutated	19 (45.2)
Wild type	23 (54.8)
BRAF**	
Mutated	3 (7.3)
Wild type	38 (92.7)

^*^Available for 42 patients. **Available for 41 patients

The median patient age was 57 years with 55.2% being female. The majority of patients with peritoneal metastases had a previous primary tumor that was T4 (67.1%).

Diagnostic laparoscopy was performed in 10 patients (14.9%) that presented a moderate-volume disease on imaging. In all these cases, a PCI score < 25 was confirmed and patients were directly candidates for CRS and HIPEC.

The majority of primary tumors were located in the right colon (53.7%).

Six patients (8.9%) received neoadjuvant chemotherapy according to irinotecan- or oxaliplatin-based systemic chemotherapy regimens.

Postoperative chemotherapy was administered to 55 (82.1%) patients.

### Resections and morbidity

Perioperative details and morbidity are shown on Tables 2 and 3.

**Table 2:** Perioperative details of patients

	No. of cases (or median) (%)
Median operation time, *min, median [range]*	318 [160–700]
ASA score	
ASA 1, *n*	12 (17.9)
ASA 2, *n*	45(67.1)
ASA 3, *n*	10 (15.0)
Length of stay, *days, median [range]*	9 [5–36]
Peritoneal carcinomatosis index (PCI)	
PCI 0–10, *n*	40 (59.7)
PCI 10–20*, n*	17 (25.3)
PCI > 20, *n*	10 (15.0)
Median PCI	7
Completeness of cytoreduction score (CC)	
CC score = 0, *n*	65 (97.0)
CC score = 2, *n*	1 (1.5)
CC score = 3, *n*	1 (1.5)
Principal resection	
Subtotal colectomy, *n*	2 (3.0)
Right hemicolectomy, *n*	33 (49.2)
Transverse colon resection, *n*	3 (4.5)
Left hemicolectomy, *n*	18 (26.9)
High rectal resection, *n*	8 (11.9)
Only peritonectomy, *n*	3 (4.5)
Associated resections, *n*	46 (68.6)
Surgical approach	
Laparotomy, *n*	53 (79.1)
Laparoscopy, *n*	14 (20.9)
Conversion rate, *n*	2 (14.2)
HIPEC technique	
Coliseum, *n*	55 (82.0)
Closed, *n*	12 (18.0)

*HIPEC* hyperthermic intraperitoneal chemotherapy

**Table 3:** Postoperative morbidity and mortality

	No. of cases (%)
Postoperative complications, *n*	24 (35.8)
Anastomotic leak	7 (10.4)
Occlusion, *n*	1 (1.5)
Bleeding, *n*	2 (3.0)
Wound infection, *n*	1 (1.5)
Lymphatic fistula, *n*	2 (3.0)
Intra-abdominal collection, *n*	1 (1.5)
Respiratory complication, *n*	4 (6.0)
Atrial fibrillation, *n*	2 (3.0)
Urinary infection, *n*	2 (3.0)
Deep vein thrombosis and pulmonary embolism, *n*	2 (3.0)
Clavien-Dindo ≥ 3	6 (8.9)
Reoperation, *n*	2 (3.0)
Postoperative mortality, *n*	0 (0)

The median PCI was 7 (range 0–32), with 57 cases (85%) having a PCI less than 20. The remaining 10 cases had a PCI more than 20, which was only determined at the time of surgery.

In our series, 64 patients presented synchronous peritoneal metastases, while the remaining 3 cases presenting metachronous peritoneal metastases underwent only peritonectomy as surgical procedure.

Hyperthermic intraperitoneal chemotherapy was given in all cases with mitomycin C, in 55 patients (82%) according to the “Coliseum” technique, and in 12 cases (18%) according to the “closed” technique.

A complete cytoreduction was achieved in 65 (97%) cases, with an incomplete cytoreduction in the remaining 2 (3%) cases.

The median length of operations was 318 min (range 160–700 min). The median length of stay was 9 days (range 5–36 days). A blood transfusion was required in approximately one-fourth of cases (23.9%). The most common procedure was right hemicolectomy (49.2%) and in 46 cases (68.6%) an associated resection was included.

Eight patients (11.9%) involved formation of a stoma.

Postoperative complication and mortality rates were 35.8% and 0%, respectively. Grade III/IV complications occurred in 6 (8.9%) cases.

There were 7 (10.4%) anastomotic leaks and 1 (1.5%) intra-abdominal collection managed with percutaneous drainage.

### Survival outcomes

Follow-up characteristics of patients are reported on Table 4.

**Table 4:** Follow-up characteristics of patients

	Patients *n* = 67 (%)
Follow-up time, *months, median [range]*	21 [0–97]
Recurrence, *n*	23 (34.3)
Loco-regional, *n*	7 (10.5)
Peritoneal, *n*	14 (20.9)
Only peritoneal, n	8 (12.0)
Distant metastases, *n*	13 (19.4)
Follow-up recurrence time, *months, median [range]*	11 [0–68]

The median follow-up for all cases was 21 months (range 2–97 months).

**Fig. 1:**
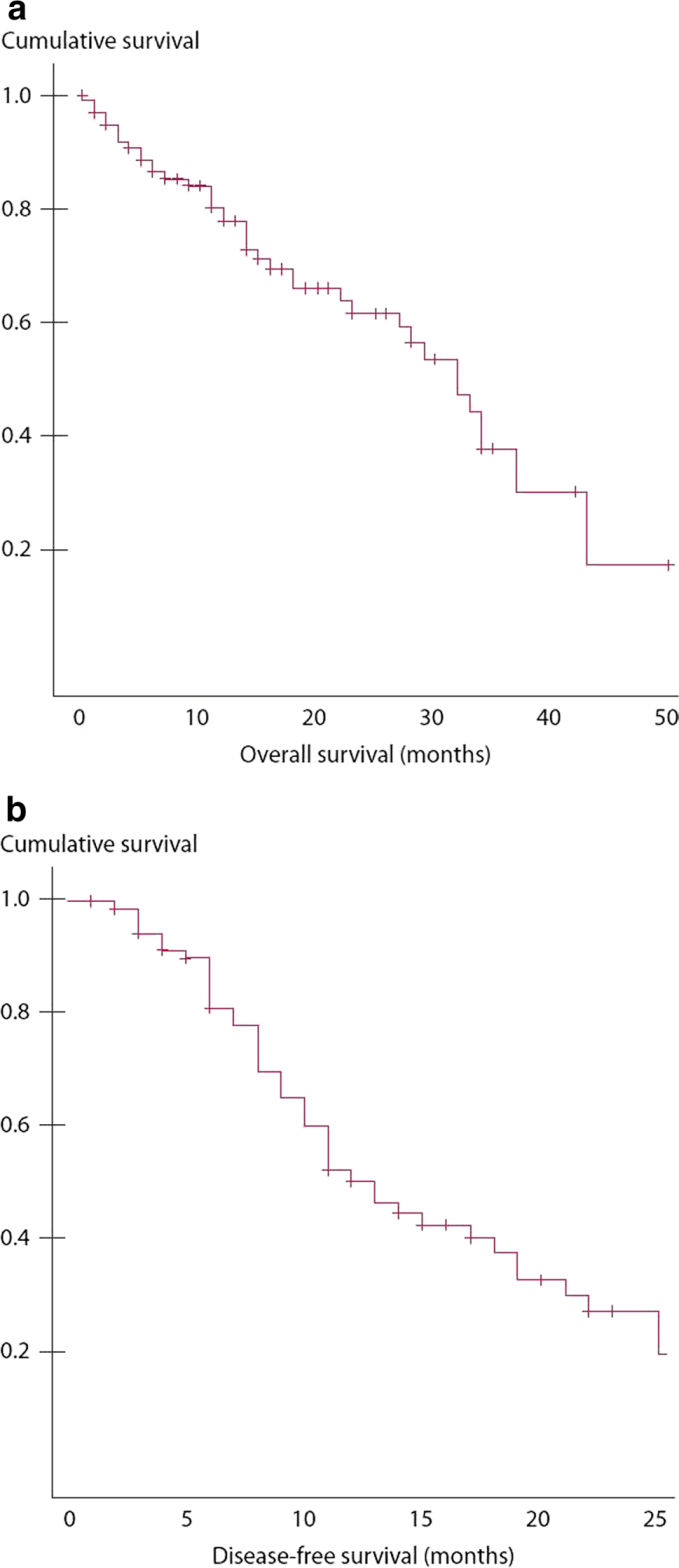
**A** OS in 67 patients after CRS plus HIPEC. **B** DFS in 65 CC0 patients after complete CRS plus HIPEC

The median survival for all cases was 41 months, with a 3-year overall survival rate of 43% (Fig. 1A). The median disease-free survival in 65 CC0 patients was 36 months (Fig. 1B).

Univariate analysis identified nonmucinous histology, PCI < 15, and completeness of cytoreduction as prognostic factors associated with improved overall survival (Table 5).

**Table 5:** Cox univariate and multivariate regression model

Variable	Univariate HR (95% CI)	*p* value	Multivariate HR (95% CI)	*p* value
Age	0.92 (0.89–1.06)	0.816	1.03 (0.99–1.06)	0.482
Sex				
Female	-			
Male	1.32 (0.75–2.42)	0.643	1.05 (0.48–2.13)	0.787
Grade III/IV complication	1.14 (0.47–2.47)	0.214	-	
Tumor site				
Colon	-			
Rectum	0.63 (0.26–1.84)	0.393		
Histology				
Adenocarcinoma	-		-	
Mucinous	4.057 (2.74–6.72)	< 0.001	3.25 (2.29–6.35)	0.003
Tumor stage				
T2	-			
T3	1.68 (0.94–2.77)	0.916		
T4	8.52 (1.25–62.40)	0.052		
Nodal stage				
N0	-			
N +	1.42 (0.53–3.07)	0.164		
RAS				
Mutated	0.38 (0.063–1.41)	0.095	0.33 (0.29–0.47)	0.24
Wild type	-			
BRAF				
Mutated	1.36 (1.25–1.63)	0.36		
Wild type	-			
Chemotherapy				
No	-		-	
Yes	0.63 (0.34–1.19)	0.130	0.69 (0.27–1.87)	0.412
CC score				
0/1	-		-	
2/3	4.52 (2.64–7.83)	< 0.001	7.58 (2.53–28.32)	0.003
PCI				
0–15	-		-	
> 15	5.35 (3.01–9.24)	< 0.001	0.79 (0.28–2.67)	0.677

*CC* completeness of cytoreduction, *PCI* peritoneal carcinomatosis index

Multivariate analysis revealed nonmucinous histology and completeness of cytoreduction as positive prognostic factors for improved overall survival (Table 5).

## Discussion

Peritoneal carcinomatosis from CRC was considered in the past as a terminal neoplastic disease and, often, it was treated only with palliative approach or best supportive care. However, during the last decade, more effective cytotoxic chemotherapies and biological targeted therapies have been developed to improve the survival of patients with metastatic disease [14, 15].

CRS and HIPEC are becoming more and more a valid approach for isolated peritoneal metastases from CRC [16].

These procedures have been applied in selected patients with CRC and peritoneal diffusion, obtaining a median survival from 12 to 32 months, and the 1-year, 2-year, 3-year, and 5-year survival rates ranging from 65 to 90%, 25 to 60%, 18 to 47%, and 17 to 30%, respectively [17].

The results of our study demonstrate an overall median survival of 39 months for the entire cohort.

Completeness of cytoreduction and nonmucinous histology were the main factors found to be independently associated with improved overall survival.

Our results are largely in accordance with other published studies.

In the first randomized controlled trial published by Verwaal et al. [11], the authors demonstrated that CRS followed by HIPEC improves survival in patients with peritoneal metastases from CRC. However, patients with an extensive involvement of the abdominal cavity, or incomplete cytoreduction, had still a poor prognosis.

Ihemelandu et al. [18] further demonstrated that for patients with a limited extent of peritoneal metastases, a complete cytoreduction is the most important prognostic variable, presenting a median survival of 36.6 months.

Other large reported series [9, 10, 19–22] have shown similar results with a median overall survival ranging from 32 to 63 months.

Huang et al. [23], in a recent meta-analysis of 76 studies, reported a median overall survival of 29 months for selected patients with peritoneal metastases from CRC.

The recently published French PRODIGE 7 randomized controlled trial [24] demonstrated that CRS alone, performed at specialized peritonectomy centers, can offer a median survival of 41.2 months with a 36.7% 5-year survival in patients with peritoneal metastases from CRC, while the addition of HIPEC with oxaliplatin does not influence the OS.

The increasing role of CRS and HIPEC for low-volume peritoneal metastasis from CRC is supported by numerous international consensus guidelines, such as the American Society of Colon and Rectal Surgeons [25] and the European Society of Medical Oncology [26].

The British National Health Service Commissioning Board includes CRS and HIPEC as part of the treatment guidelines for patients with limited peritoneal metastases from CRC [27].

The Australian Cancer Council recommends referral to a specialized center in peritoneal surface malignancies for consideration of CRS and HIPEC in case of patients with low-volume peritoneal involvement [28].

Unfortunately, this multimodal procedure is burdened by higher or similar morbidity and mortality rates with respect to other major gastrointestinal interventions [29].

A mortality rate ranging from 0.9 to 11% [13, 28–30] and a major morbidity rate that ranges from 12 to 57% in high-volume centers were reported [28–31] in the literature, even if, in recent publications, overall grade III–IV morbidity rates is decreased between 7 and 41% [32–38]. These results are strictly related to the improvement of the experience of specialized centers in peritoneal surface malignancies [39, 40].

Kusamura et al. [33] demonstrated in their manuscript, on 209 peritoneal surface malignancies treated with closed HIPEC, a morbidity and mortality rate of 12% and 0.9%, respectively. On multivariate analysis, extent of cytoreduction and dose of intraperitoneal cisplatin were independent prognostic factors for major morbidity.

In the French study conducted by Glehen et al. [34] on 216 consecutive procedures, the authors found that morbidity was significantly related to the carcinomatosis stage (*p* = 0.016), the duration of surgery (*p* = 0.005), and the number of resections and peritonectomy procedures (*p* = 0.042).

High morbidity and mortality rates associated to CRS and HIPEC were also observed in the multivariate analysis conducted by Hansson et al. on 123 patients [35]. But, considering the potential benefit on long-term outcomes, the possible negative events are considered acceptable.

In the multivariate analysis conducted by Casado et al. [36] on 147 consecutive patients with peritoneal surface malignancy treated by CRS and HIPEC, only PCI was identified as a negative prognostic factor for gastrointestinal complications (*p* = 0.058). Moreover, the frequency of gastrointestinal complications was associated with a large extent of disease, according to a PCI > 30.

Finally, the Japanese study by Mizumoto et al. [37] showed a global morbidity rate of 49%, demonstrating that PCI greater than 20 was the only significant risk factor for postoperative complications (*p* < 0.01), whereas HIPEC significantly reduced postoperative complications (*p* < 0.05).

These results are worse than those achieved in our study. In fact, we recorded a postoperative morbidity rate of 35.8%, with a rate of grade 3–4 complications of 8.9%, while the mortality rate was 0%.

A number of prognostic factors have been found to be associated with overall survival. We found nonmucinous histology and completeness of cytoreduction to be independently associated with improved overall survival.

Other studies have found the completeness of cytoreduction, PCI, positive lymph nodes, histology, use of systemic perioperative chemotherapy, and the experience of the center to be prognostic factors influencing overall survival [9, 10, 41–43].

## Conclusions

Our study presents a few limitations that needs to be discussed.

First of all, due to its retrospective nature, it is strictly dependent on the accuracy of prerecorded data and the accuracy of patients’ selection. For these reasons, all patient records were thoroughly reviewed to ensure data accuracy.

Moreover, this also highlights the important limitations in current staging modalities.

Unfortunately, imaging methods such as CT and PET/CT scans offer a sensitivity in the detection of peritoneal metastases that can reach, but not overcome, 90% [44, 45].

Moreover, an important and significant challenge remains the ability to accurately predict a preoperative PCI, thus allowing the possibility to reach a complete cytoreduction.

Moreover, in the future, only an extensive diffusion of routine staging laparoscopy may help to better select patients for CRS and HIPEC.

In conclusion, CRS and HIPEC can offer long-term survival for selected patients with limited peritoneal metastasis from CRC, with acceptable morbidity and mortality rates. The CC score and histology (nonmucinous) represent the most important prognostic factors associated with improved long-term outcomes.

## References

[1] Bray F, Ferlay J, Soerjomataram I (2018). A Global Cancer Statistics 2018: GLOBOCAN estimates of incidence and mortality worldwide for 36 cancers in 185 countries. CA Cancer J Clin.

[2] Franko J, Shi Q, Meyers JP (2016). Analysis and Research in Cancers of the Digestive System (ARCAD) Group, Prognosis of patients with peritoneal metastatic colorectal cancer given systemic therapy: an analysis of individual patient data from prospective randomised trials from the Analysis and Research in Cancers of the Digestive System (ARCAD) database. Lancet Oncol.

[3] Klaver CE, Groenen H, Morton DG (2017). research committee of the European Society of Coloproctology, Recommendations and consensus on the treatment of peritoneal metastases of colorectal origin: a systematic review of national and international guidelines. Colorectal Dis.

[4] Elferink MA, de Jong KP, Klaase JM (2015). Metachronous metastases from colorectal cancer: a population based study in North-East Netherlands. Int J Colorectal Dis.

[5] Sadeghi B, Arvieux C, Glehen O (2000). Peritoneal carcinomatosis from non-gynecologic malignancies: results of the EVOCAPE 1 multicentric prospective study. Cancer.

[6] Sugarbaker PH (1999). Management of peritoneal-surface malignancy: the surgeon’s role. Langenbeck’s Arch Surg.

[7] Sugarbaker PH (2006). New standard of care for appendiceal epithelial neoplasms and pseudomyxoma peritonei syndrome?. Lancet Oncol.

[8] Sugarbaker PH (1995). Peritonectomy procedures. Ann Surg.

[9] Glehen O, Kwiatkowski F, Sugarbaker PH (2004). Cytoreductive surgery combined with perioperative intraperitoneal chemotherapy for the management of peritoneal carcinomatosis from colorectal cancer: a multi-institutional study. J Clin Oncol.

[10] Elias D, Lefevre JH, Chevalier J (2009). Complete cytoreductive surgery plus intraperitoneal chemohyperthermia with oxaliplatin for peritoneal carcinomatosis of colorectal origin. J Clin Oncol.

[11] Verwaal VJ, van Ruth S, de Bree E (2003). Randomized trial of cytoreduction and hyperthermic intraperitoneal chemotherapy versus systemic chemotherapy and palliative surgery in patients with peritoneal carcinomatosis of colorectal cancer. J Clin Oncol.

[12] Esquivel J, Elias D, Baratti D (2008). Consensus statement on the loco regional treatment of colorectal cancer with peritoneal dissemination. J Surg Oncol.

[13] Dindo D, Demartines N, Clavien P-A (2004). Classification of surgical complications. Ann Surg.

[14] Marz L, Piso P (2015). Treatment of peritoneal metastases from colorectal cancer. Gastroenterol Rep.

[15] Macrì A, Arcoraci V, Belgrano V (2020). Short-term outcome of cytoreductive surgery and hyperthermic intraperitoneal chemotherapy used as treatment of colo-rectal carcinomatosis: a multicentric study. Updat Surg.

[16] Narasimhan V, Britto M, Pham T (2019). Evolution of cytoreductive surgery and hyperthermic intraperitoneal chemotherapy for colorectal peritoneal metastases: 8-year single-institutional experience. Dis Colon Rectum.

[17] Macrì A, Saladino E, Bartolo V (2010). Peritoneal carcinomatosis of colorectal origin. World J Gastrointest Oncol.

[18] Ihemelandu C, Sugarbaker PH (2017). Management for peritoneal metastasis of colonic origin: role of cytoreductive surgery and perioperative intraperitoneal chemotherapy: a single institution’s experience during two decades. Ann Surg Oncol.

[19] Alzahrani N, Ferguson JS, Valle SJ (2016). Cytoreductive surgery and hyperthermic intraperitoneal chemotherapy: long-term results at St George Hospital, Australia. ANZ J Surg.

[20] Esquivel J (2016). Cytoreductive surgery and hyperthermic intraperitoneal chemotherapy for colorectal cancer: survival outcomes and patient selection. J Gastrointest Oncol.

[21] Cashin PH, Graf W, Nygren P (2012). Cytoreductive surgery and intraperitoneal chemotherapy for colorectal peritoneal carcinomatosis: prognosis and treatment of recurrences in a cohort study. Eur J Surg Oncol.

[22] Glehen O, Gilly FN, Boutitie F (2010). French Surgical Association, Toward curative treatment of peritoneal carcinomatosis from nonovarian origin by cytoreductive surgery combined with perioperative intraperitoneal chemotherapy: a multi-institutional study of 1,290 patients. Cancer.

[23] Huang CQ, Min Y, Wang SY (2017). Cytoreductive surgery plus hyperthermic intraperitoneal chemotherapy improves survival for peritoneal carcinomatosis from colorectal cancer: a systematic review and meta-analysis of current evidence. Oncotarget.

[24] Quénet F, Elias D, Roca L (2021). UNICANCER-GI Group and BIG Renape Group, Cytoreductive surgery plus hyperthermic intraperitoneal chemotherapy versus cytoreductive surgery alone for colorectal peritoneal metastases (PRODIGE 7): a multicentre, randomised, open-label, phase 3 trial. Lancet Oncol.

[25] Vogel JD, Eskicioglu C, Weiser MR (2017). The American Society of Colon and Rectal Surgeons clinical practice guidelines for the treatment of colon cancer. Dis Colon Rectum.

[26] Van Cutsem E, Cervantes A, Adam R (2016). ESMO consensus guidelines for the management of patients with metastatic colorectal cancer. Ann Oncol.

[27] NHS Commissioning Board: Clinical Commissioning Policy for Cytoreductive Surgery with Hyperthermic Intraperitoneal Chemotherapy for Peritoneal Carcinomatosis. NHSCB/A08/P/a April 2013. www.england.nhs.uk/wp-content/uploads/2013/09/a08-p-a.pdf

[28] Luck A, Koh C, Chow M, Chetcuti A, COLMNG3: What is the role for peritonectomy with or without PIC in the treatment recurrent as well as primary colorectal cancer with peritoneal involvement (not including appendiceal neoplasia)? Sydney: Cancer Council Australia. Available at: https://wiki.cancer.org.au/australia/Guidelines:Colorectal_cancer (access date: 31/05/2021)

[29] Chua TC, Yan TD, Saxena A (2009). Should the treatment of peritoneal carcinomatosis by cytoreductive surgery and hyperthermic intraperitoneal chemotherapy still be regarded as a highly morbid procedure? A systematic review of morbidity and mortality. Ann Surg.

[30] Jaquet P, Sugarbaker PH (1996). Clinical research methodologies in diagnosis and staging of patients with peritoneal carcinomatosis. Cancer Treat Res.

[31] Stewart JH, Shen P, Levine EA (2008). Intraperitoneal hyperthermic chemotherapy: an evolving paradigm for the treatment of peritoneal surface malignancies. Expert Rev Anticancer Ther.

[32] Saladino E, Fleres F, Mazzeo C (2014). The role of prophylactic hyperthermic intraperitoneal chemotherapy in the management of serosal involved gastric cancer. Anticancer Res.

[33] Kusamura S, Younan R, Costanzo Baratti D (2006). Cytoreductive surgery followed by intraperitoneal hyperthermic perfusion: analysis of morbidity and mortality in 209 peritoneal surface malignancies treated with closed abdomen technique. Cancer.

[34] Glehen O, Osinsky D, Cotte E (2003). Intraperitoneal chemohyperthermia using a closed abdominal procedure and cytoreductive surgery for the treatment of peritoneal carcinomatosis: morbidity and mortality analysis of 216 consecutive procedures. Ann Surg Oncol.

[35] Hansson J, Graf W, Pahlman L (2009). Postoperative adverse events and long-term survival after cytoreductive surgery and intraperitoneal chemotherapy. Eur J Surg Oncol.

[36] Casado-Adam A, Alderman R, Stuart OA (2011). Gastrointestinal complications in 147 consecutive patients with peritoneal surface malignancy treated by cytoreductive surgery and perioperative intraperitoneal chemotherapy. Int J Surg Oncol.

[37] Mizumoto A, Canbay E, Hirano M (2012). Morbidity and mortality outcomes of cytoreductive surgery and hyperthermic intraperitoneal chemotherapy at a single institution in Japan. Gastroenterol Res Pract.

[38] Di Giorgio A, De Iaco P, De Simone M (2017). Cytoreduction (peritonectomy procedures) combined with hyperthermic intraperitoneal chemotherapy (HIPEC) in advanced ovarian cancer: retrospective Italian multicenter observational study of 511 cases. Ann Surg Oncol.

[39] Smeenk RM, Verwaal VJ, Zoetmulder FA (2007). Learning curve of combined modality treatment in peritoneal surface disease. Br J Surg.

[40] Kusamura S, Baratti D, Deraco M (2012). Multidimensional analysis of the learning curve for cytoreductive surgery and hyperthermic intraperitoneal chemotherapy in peritoneal surface malignancies. Ann Surg.

[41] Piso P, Stierstorfer K, Gerken M (2018). Benefit of cytoreductive surgery combined with hyperthermic intraperitoneal chemotherapy in patients with isolated peritoneal metastases from colorectal cancer. Int J Colorectal Dis.

[42] Elias D, Gilly F, Boutitie F (2010). Peritoneal colorectal carcinomatosis treated with surgery and perioperative intraperitoneal chemotherapy: retrospective analysis of 523 patients from a multicentric French study. J Clin Oncol.

[43] de Cuba EM, Kwakman R, Knol DL (2013). Cytoreductive surgery and HIPEC for peritoneal metastases combined with curative treatment of colorectal liver metastases: systematic review of all literature and meta-analysis of observational studies. Cancer Treat Rev.

[44] Chang MC, Chen JH, Liang JA (2013). PET or PET/CT for detection of peritoneal carcinomatosis: a meta-analysis. Clin Nucl Med.

[45] Kim SJ, Lee SW (2018). Diagnostic accuracy of 18F-FDG PET/CT for detection of peritoneal carcinomatosis; a systematic review and meta-analysis. Br J Radiol.

